# Rice Bran Oil Improves Emphysema in Cigarette Smoke Extract-Induced Mice through Anti-Inflammatory and Antioxidative Effects

**DOI:** 10.3390/nu16030433

**Published:** 2024-01-31

**Authors:** Aikkarach Kettawan, Sukpattaraporn Ruangklai, Thanaporn Rungruang, Julalux Thongam, Aurawan Kringkasemsee Kettawan, Nilesh Nirmal, Sorachai Srisuma

**Affiliations:** 1 Institute of Nutrition, Mahidol University, Nakhon Pathom 73170, Thailand; aikkarach.ket@mahidol.ac.th (A.K.); aurawan.kri@mahidol.ac.th (A.K.K.); nilesh.nir@mahidol.ac.th (N.N.); 2 Department of Physiology, Faculty of Medicine Siriraj Hospital, Mahidol University, Bangkok 10700, Thailandjulalux.tho@mahidol.ac.th (J.T.); 3 Department of Anatomy, Faculty of Medicine Siriraj Hospital, Mahidol University, Bangkok 10700, Thailand; thanaporn.run@mahidol.ac.th

**Keywords:** cigarette smoke extract, lung inflammation, emphysema, rice bran oil, antioxidant, anti-inflammatory, health

## Abstract

Lung inflammation and alveolar enlargement are the major pathological conditions of chronic obstructive pulmonary disease (COPD) patients. Rice bran oil (RBO), a natural anti-inflammatory and antioxidative agent, has been used for therapeutic purposes in several inflammatory diseases. This study aimed to investigate the anti-inflammatory and antioxidative effect of RBO on a cigarette smoke extract (CSE)-induced emphysema model in mice. The results indicated that CSE significantly induced airspace enlargement in mouse lung. Increased inflammatory cells, macrophage, and TNF-alpha levels in bronchoalveolar lavage fluid (BALF) were noticed in CSE-treated mice. RBO (low and high dose)-supplemented mice showed decreased total BALF inflammatory cell, macrophage, and neutrophil numbers and TNF-alpha levels (*p* < 0.05). Additionally, the administration of RBO decreased the mean linear alveolar intercept (MLI) in the CSE-treated group. Additionally, RBO treatment significantly increased the total antioxidant capacity in both mouse BALF and serum. However, RBO did not have an effect on the malondialdehyde (MDA) level. These findings suggested that RBO treatment ameliorates lung inflammation in a CSE-induced emphysema mice model through anti-inflammatory and antioxidant pathways. Therefore, the supplementation of RBO could be a new potential therapeutic to relieve the severity of COPD.

## 1. Introduction

Chronic obstructive pulmonary disease (COPD) is a chronic inflammatory lung disease presented by persistent airflow obstruction and is a primary cause of global comorbidity and mortality [1]. Emphysema is one of the major pathological features of COPD, characterized by enlarged airspace due to the destruction of alveolar walls [2]. The global prevalence of COPD is increased every year, with the age of patients presenting with the disease decreasing age worldwide [3]. There were 480 million cases in 2020 [4]. The World Health Organization (WHO) reported that COPD is the third leading cause of death worldwide, with 3 million deaths in 2019, and is responsible for 6% of total global deaths, after ischemic heart disease and stroke [5]. While COPD is not currently curable, its symptoms can be effectively managed through medical therapies [6]. Therefore, effective treatment of COPD is necessary concerning the causative factors and pathology of COPD [7].

The most prevalent risk factor for COPD development is reported to be exposure to cigarette smoke, leading to abnormal inflammation in the lung [8]. The main characteristic of COPD is chronic airway obstruction causing airflow limitation, increased airway resistance, and gas exchange abnormality in patients. The toxic particles in cigarette smoke induce an inflammatory response in the lung, resulting in a decreased diameter of the airway and initiating airflow obstruction. Noxious particles in a cigarette can directly damage epithelial cells in the lung [9]. Damaged epithelial cells release proinflammatory cytokines (e.g., tumor necrosis factor (TNF)-alpha) to recruit inflammatory cells into the location of the event. Inflammatory cells such as monocytes, neutrophils, and lymphocytes are recruited and accumulated in the lung. Accumulation of inflammatory cells participates in inflammation by releasing pro-inflammatory cytokines including interleukin (IL)-6, IL-8, and TNF-alpha to promote inflammation [8]. In addition, inflammatory cells can release protease and reactive oxygen species (ROS) to enhance inflammatory response in the lung [10,11]. Abnormal lung inflammation continuously causes the release of pro-inflammatory cytokines, protease, and ROS as a vicious cycle promoting progressive airflow obstruction. Additionally, oxidative stress has been implicated in the pathogenesis of COPD and is a major mechanism in the progression of severity in COPD patients [9]. Thus, boosting endogenous levels of antioxidants and inhibiting inflammatory response are probably advantageous in the treatment of COPD [12]. Previous studies reported fewer exacerbations after treatment with N-acetyl-L-cysteine (NAC), an antioxidant and anti-inflammatory drug, in COPD patients [13]. However, NAC creates the risk of bronchospasm in hyperreactive patients [14]. Inhaled medicines including bronchodilators and corticosteroids are treatments available for COPD. Corticosteroids, one of the anti-inflammatory drugs, have several side effects after prolonged use. As a result, there are quite limited pharmaceutical approaches for the treatment of COPD [15]. Therefore, the investigation of novel antioxidant and anti-inflammatory compounds remains an important area of research for COPD treatment.

Rice bran oil (RBO) is a natural antioxidative and anti-inflammatory supplement consisting of gamma-oryzanol, tocopherols, and tocotrienols [16]. Especially, gamma-oryzanol is a natural bioactive phytochemical with well-known antioxidative and anti-inflammatory activities [17]. A previous study reported that RBO protected cells from a free radical reaction [18]. Also, RBO was demonstrated to have an anti-inflammatory ability by decreasing proinflammatory cytokines and suppressing NF-kB activity on activated macrophages isolated from the rat peritoneal cavity [19]. Moreover, several studies revealed that RBO has been used as a therapeutic approach in many inflammatory and oxidative stress disorders, including diabetes, liver cirrhosis, hypercholesterolemia, cancer, and rheumatism in rat/mouse models [16,20,21,22,23,24].

However, the anti-inflammatory and antioxidative effect of RBO in the emphysema mouse model has not been reported. C57BL/6 mice are one of the standard animal models for studying mechanisms of emphysema. There are at least two ways to induce emphysema in mouse models, including exposure to cigarette smoke (CS) or cigarette smoke extract (CSE) [25]. The previous research examined the effect of CS- and CSE-induced emphysema in eight-week-old male C57BL/6 mice compared with the control group [26]. The findings revealed that 12-week CS inhalation and 4-week CSE intraperitoneal injection significantly increased inflammation, alveolar degradation, and airspace enlargement in mouse lungs when compared with their control. Importantly, there was no statistically significant difference among all those parameters between the CS and CSE groups [26]. Intraperitoneal CSE injection has been used to induce pulmonary emphysema in rat and mouse models [27,28,29]. Therefore, CSE-exposed C57BL/6 mice were used as an emphysema mouse model for this study. This study investigated the anti-inflammatory and antioxidative effects of RBO supplementation after intraperitoneal CSE administration in cigarette smoke extract (CSE)-induced emphysema mice.

## 2. Materials and Methods

### 2.1. Cigarette Smoke Extract (CSE) Preparation 

Cigarette smoke extract (CSE) solution was prepared as described [26]. Briefly, one Marlboro Red cigarette with a filter (Marlboro, Philip Morris, New York, NY, USA) was burned in a plastic box. The mainstream of smoke was suctioned by a vacuum pump and passed through 1 mL of phosphate-buffered saline (PBS) solution for collecting CSE. The CSE solution’s pH was adjusted to 5.2–5.4. Then, the CSE solution was filtered through 0.22 μm pores (Thermo Fisher Scientific, Waltham, MA, USA) to remove particles. The solution was freshly prepared under a fume hood for each experiment.

### 2.2. Animal Experiment 

C57BL/6 mice (male; age 6–8 weeks; weight 20 ± 2 g) were purchased from Nomura Siam International Co., Ltd., Bangkok, Thailand. Mice were acclimatized and accommodated in standard cages (5 mice/cage) within controlled room conditions of 23 ± 1 °C and 50 ± 10% humidity on a 12 h light and dark cycle. Food and water were supplied for all the mice. The methods used for the animal experiment were authorized by the Siriraj Animal Care and Use Committee, Mahidol University (SI-ACUP no. 012/2562).

**Figure 1:**
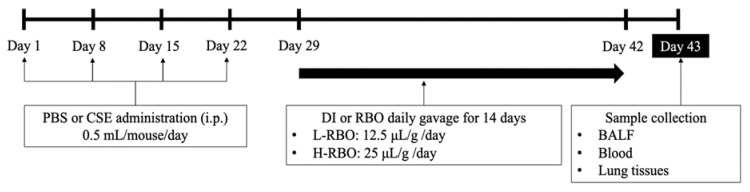
Experimental design of the study on effect of RBO on CSE-induced emphysema mouse model. PBS, phosphate-buffered saline; CSE, cigarette smoke extract; DI, distilled water; RBO, rice bran oil; L-RBO, low dose of rice bran oil; H-RBO, high dose of rice bran oil; i.p., intraperitoneal injection; BALF, bronchoalveolar lavage fluid.

RBO containing 12,000 ppm gamma-oryzanol (on the label) was purchased from the supermarket in Bangkok. Our analysis results of gamma-oryzanol content by the HPLC method [30] was equal to 12,100 ± 150 ppm (*n* = 3 bottles). The entire trial lasted six weeks. On days 1, 8, 15, and 22, mice in the control group were given PBS at a dose of 0.5 mL/mouse via intraperitoneal injection (i.p.), and mice in the emphysema-induced group were given CSE at a dose of 0.5 mL/mouse i.p. (Figure 1). Each group was further randomly separated into three subgroups (10 mice per group) including distilled water (DI), RBO at a low dose (L-RBO), and RBO at a high dose (H-RBO). Therefore, there were a total of six experimental groups (1) PBS+DI, (2) PBS+L-RBO, (3) PBS+H-RBO, (4) CSE+DI, (5) CSE+L-RBO, and (6) CSE+H-RBO, with 7–10 mice for each group. On day 29, mice in groups 2, 3, 5, and 6 received gavage feeding with an RBO dosage of 12.5 μL/g body weight (for low-dose groups) and 25 μL/g body weight (for high-dose groups) daily for 14 days, while the mice in groups 1 and 4 received only DI. The dosage of RBO would provide the equivalent intake in an adult human of 0.92 g and 1.84 g/kg body weight, respectively, when calculated from the body weight and body surface area of mice [31]. The mice’s body weight and food consumption were monitored weekly during the investigation. After treatment (day 43), all mice were anesthetized and euthanized by CO_2_ asphyxiation and cervical dislocation.

### 2.3. Sample Collection

Blood was collected by cardiac puncture and kept in a −80 °C refrigerator for further investigation. Bronchoalveolar lavage fluid (BALF) was collected by pushing 1 mL of normal saline solution (NSS) into the lung, and then, the lung fluid was gently drawn back and collected in microcentrifuge tubes. After heart perfusion, the right lung was clamped, and the left lung was inflated with 10% low-melting-temperature agarose (Invitrogen, Carlsbad, CA, USA) dissolved in a sterile normal saline solution (Terumo, Tokyo, Japan) into the trachea at a transpulmonary pressure of 25–30 cm H_2_O, monitored by pressure manometer (Thermo Fisher Scientific Inc., Waltham, MA, USA). Then, the inflated lung was removed and fixed in 10% buffered formalin (Vidhyasom Co., Ltd., Bangkok, Thailand) for 24 h to examine histopathology of the lung. The right lung was removed and kept in −80 °C refrigerator for further biochemical analysis.

### 2.4. Total and Differential Cell Count in Bronchoalveolar Lavage Fluid (BALF)

The BALF was immediately centrifuged for 10 min at 1500 rpm at 4 °C. Total cells were isolated for counting and stained with 0.4% trypan blue (Thermo Fisher Scientific Inc., Waltham, MA, USA) with a dilution of 1:1. The total cell count was operated using Bright-Line hemocytometer (Hausser Scientific, Horsham, PA, USA) and manually counted under a light microscope at 40× magnification.

For differential cell count, 0.3 mL of BALF was centrifuged at 650 rpm for 10 min in a cytocentrifuge chamber (Thermo Fisher Scientific Inc., Waltham, MA, USA). Cells on the glass slide were fixed by dipping in methanol and immediately dipping in eosin solution. Then, the glass slide was stained with 0.5% methylene blue for 4 min. Differential cell count was performed using a Bright-Line hemocytometer and identified using a light microscope at a magnification of 100×. The percentages of alveolar macrophages, neutrophils, eosinophils, and lymphocytes were analyzed.

### 2.5. Lung Histopathological Examination

The formalin-fixed lung tissue was midsagittally sectioned, embedded in paraffin, and cut and stained with hematoxylin and eosin (H&E), followed by the standard staining procedure. Finally, the sections were mounted using Canada balsam reagent (Sigma-Aldrich Pte. Ltd., Singapore). The whole lung section was visualized under Photoshop CS5 (version 12.0) and imaged at 20× magnification under Olympus BX43 light microscope (Olympus Corporation, Tokyo, Japan). Ten areas from each one whole lung sectioned image were randomly selected for the mean linear intercept (MLI) analysis. MLI represents the distance between consecutive intersections of alveolar walls as quantitative data to determine airspace enlargement in the lung [32]. The MLI was determined by dividing the length of a line drawn across the lung section by the total number of alveolar intercepts counted inside this line. The histomorphology assessment was performed in a blinded manner.

### 2.6. Cytokines Analysis in BALF

The BALF sample was centrifuged at 4 °C for 10 min, and then the supernatant of BALF was collected. The level of TNF-alpha was determined using a sandwich enzyme-linked immunosorbent assay (ELISA) kit (Boster Biological Technology, Pleasanton, CA, USA), according to the manufacturer’s instructions.

### 2.7. Lipid Peroxidation Estimation

The supernatant of BALF was collected after centrifugation at 4 °C for 10 min. The level of malondialdehyde (MDA) was determined using a sandwich enzyme-linked immunosorbent assay (ELISA) kit (Boster Biological Technology, Pleasanton, CA, USA), according to the manufacturer’s instructions.

### 2.8. Oxygen Radical Absorbance Capacity (ORAC) Analysis

BALF and serum samples were used to analyze anti-oxidant capacity in the emphysema mouse model. The BALF samples were centrifuged at 1500 rpm for 60 min and the blood samples were centrifuged at 3500 rpm for 20 min at 4 °C. The supernatant (500 μL) was collected and mixed with 3 mL of fluorescein working solution and incubated in 37 °C water bath. Then, the mixture was mixed with 500 μL of 2,2′-azobis (2-amidinopropane) di-hydrochloride (AAPH) (Sigma-Aldrich Pte. Ltd., Singapore). Trolox (6-hydroxy-2,5,7,8-tetremethychroman-2-carboxylic acid) (Sigma-Aldrich Pte. Ltd., Singapore) (6.25–100 µM) served as a standard. Blank, a negative control, contained 500 μL of ORAC buffer working solution. The absorbance of Trolox standard, samples, and blank was determined an excitation wavelength at 493 nm and an emission wavelength at 515 nm using a spectrofluorometer. The intensity of fluorescein was measured every 1 min until the value appeared nearly constant or equal to zero. The value between the sample and blank was calculated of the area under the curve (AUC) and net area under the curve (net AUC) for comparison with the standard curve of the Trolox standard. The value was presented in µM of Trolox per mL samples.

### 2.9. Statistical Analysis

All parameters were tested to determine the normality of the data by using the normal distribution. Then, the data were analyzed by unpaired T-test (independent T-test) after comparing with age-matched control groups. To evaluate the differences among groups, one-way ANOVA with Bonferroni’s post hoc test was used. The results were presented as mean ± standard error of the mean (SEM). A *p*-value less than 0.05 was considered statistically significant.

## 3. Results

### 3.1. Body Weight and Weight Gain in CSE-Induced Emphysema in Mice 

**Figure 2:**
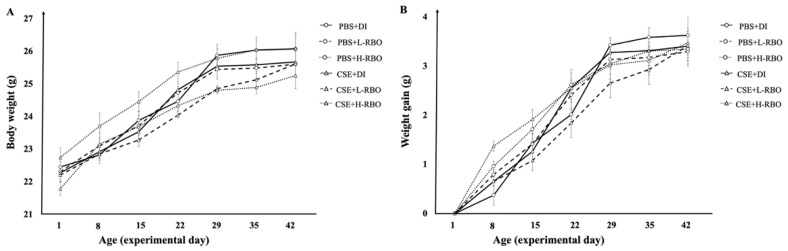
Effect of RBO on body weight (**A**) and weight gain (**B**) of mouse PBS (circle) and CSE (triangle) group. Solid, dashed, and dotted lines represent water, low, and high dose of RBO. All values are presented as mean ± SEMs (*n* = 10). One-way ANOVA (Bonferroni’s post hoc test) was used to evaluate the differences among groups. PBS, phosphate-buffered saline; CSE, cigarette smoke extract; DI, distilled water; L-RBO, low dose of rice bran oil; H-RBO, high dose of rice bran oil.

At baseline, there was no significant difference in the body weight of the CSE-induced groups when compared with the PBS-induced groups (*p* > 0.05). The body weight increased in all mice throughout the study (Figure 2A). In addition, there was no significant difference in weight gain of PBS-induced groups compared to the CSE-induced groups on days 8, 15, 22, 29, 35, and 42 (*p* > 0.05) (Figure 2B). The results suggested that RBO did not affect the body weight in the PBS-induced groups or CSE-induced emphysema in mice.

### 3.2. Effect of RBO on Lung Morphology and Airspace Size in CSE-Induced Emphysema in Mice 

**Figure 3:**
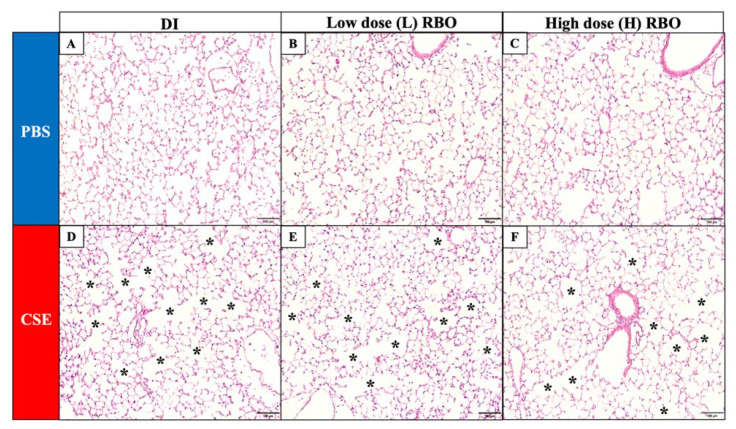

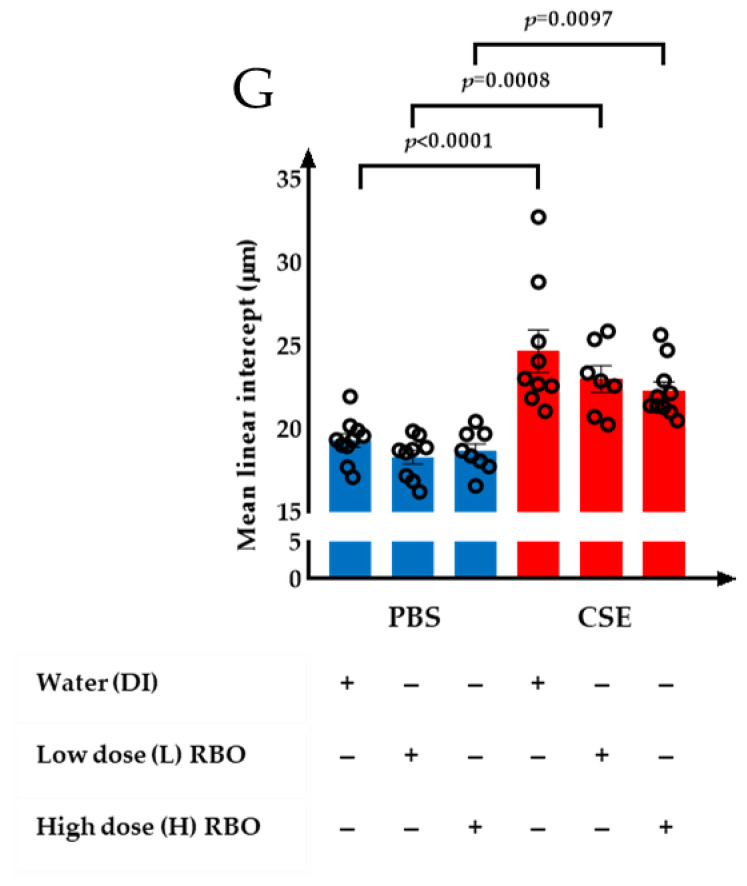
Effect of RBO on lung histomorphology and airspace size. Representative images of lung tissue section stained with hematoxylin and eosin (H&E) of PBS (**A**–**C**) and CSE groups (**D**–**F**) at a magnification of 200× under a microscope. Scale bars represent 50 μm. A black asterisk indicates an area with reduced alveolar tissue. (**G**) Mean linear intercept of mouse PBS and CSE groups. All values are presented as mean ± SEMs (*n* = 7–10). One-way ANOVA (Bonferroni’s post hoc test) was used to evaluate the differences among groups with *p*-values for each pair-wise comparison. PBS, phosphate-buffered saline; CSE, cigarette smoke extract; DI, distilled water; L-RBO, low dose of rice bran oil; H-RBO, high dose of rice bran oil.

Enlarged airspace was predominantly observed in the microscopic structure of mouse lung in the CSE+DI group (Figure 3D) when compared with the PBS+DI (Figure 3A). For quantitatively morphometric changes in mouse lung, the CSE-induced groups significantly increased MLI in all groups when compared with the PBS-induced groups (*p* < 0.01) (Figure 3G). Compared to the CSE+DI group, the CSE+L-RBO group (Figure 3E) and CSE+H-RBO group (Figure 3F) did not have any significant effect on MLI (*p* > 0.999 and *p* = 0.255, respectively). Furthermore, there was no significant difference in MLI among three of the PBS-induced groups (PBS+DI; 19.34 ± 0.27 μm vs. PBS+L-RBO (Figure 3B); 18.35 ± 0.27 μm vs. PBS+H-RBO (Figure 3C); 18.83 ± 0.30 μm, *p* = 0.178).

### 3.3. Effect of RBO on Inflammatory Cells in CSE-Induced Emphysema in Mice

**Figure 4:**
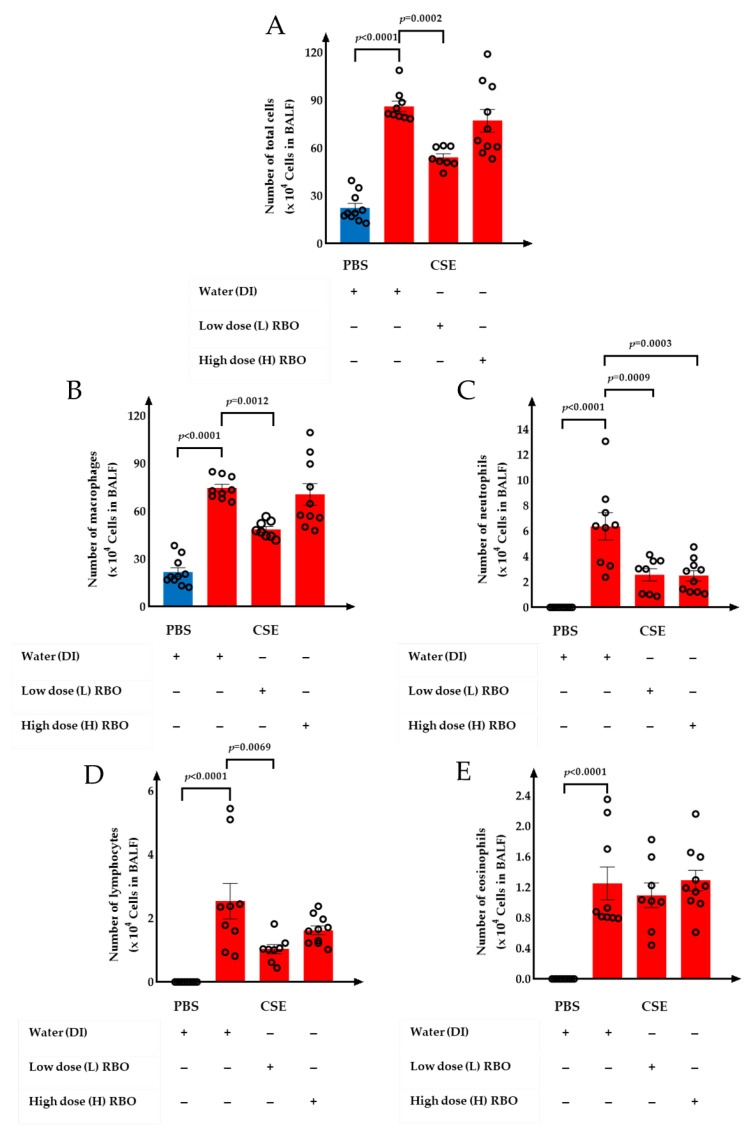
Effect of RBO on the number of (**A**) total cells, (**B**) macrophages, (**C**) neutrophils, (**D**) lymphocytes, and (**E**) eosinophils in the BALF sample. All values are presented as mean ± SEMs (*n* = 8–10). One-way ANOVA (Bonferroni’s post hoc test) was used to evaluate the differences among groups with *p*-values for each pair-wise comparison. PBS, phosphate-buffered saline; CSE, cigarette smoke extract; DI, distilled water; L-RBO, low dose of rice bran oil; H-RBO, high dose of rice bran oil; BALF, bronchoalveolar lavage fluid.

To examine the anti-inflammatory effect of RBO on CSE-induced emphysema in mice, the number of total inflammatory cells and differential cell types in BALF samples was counted (Figure 4). The CSE+DI group showed a significant increase in the number of total inflammatory cells, macrophages, neutrophils, lymphocytes, and eosinophils when compared with the PBS+DI group (*p* < 0.001). In comparison with the CSE+DI group, a low dose of RBO supplement (CSE+L-RBO) significantly decreased the number of total inflammatory cells, macrophages, neutrophils, and lymphocytes in the BALF sample (*p =* 0.002), while a high dose of RBO supplement (CSE+H-RBO) showed a significant decrease only in the number of neutrophils (*p* = 0.003). However, low and high doses of RBO supplements did not have a significant effect on the number of eosinophils (*p* = 1.00). The number of total inflammatory cells, macrophages, neutrophils, lymphocytes, and eosinophils in the CSE+L-RBO and CSE+H-RBO groups, however, was significantly higher than the PBS groups (*p* < 0.001).

### 3.4. Effect of RBO on the Levels of TNF-Alpha in CSE-Induced Emphysema in Mice 

**Figure 5:**
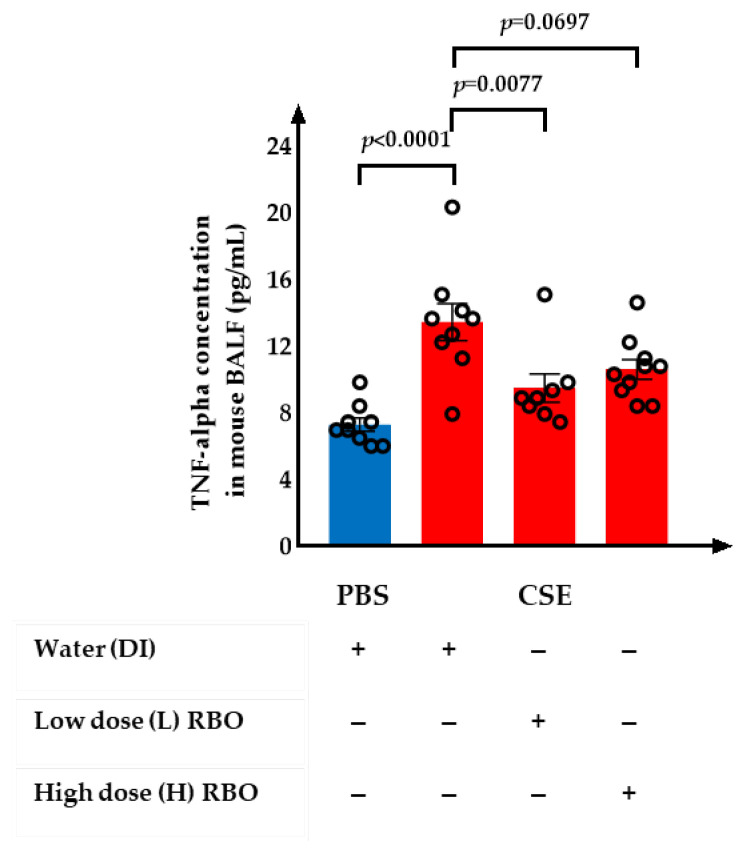
Effect of RBO on the levels of TNF-alpha in mouse BALF sample. All values are presented as mean ± SEMs (*n* = 8–10). One-way ANOVA (Bonferroni’s post hoc test) was used to evaluate the differences among groups with *p*-values for each pair-wise comparison. PBS, phosphate-buffered saline; CSE, cigarette smoke extract; DI, distilled water; L-RBO, low dose of rice bran oil; H-RBO, high dose of rice bran oil; BALF, bronchoalveolar lavage fluid.

The levels of TNF-alpha in the mouse BALF samples are shown (Figure 5). The CSE-induced group had significantly increased TNF-alpha levels compared to the PBS-induced group (*p* < 0.001). The supplementation of L-RBO showed significant inhibition of TNF-alpha levels (*p* = 0.0077), whereas H-RBO tended to decrease TNF-alpha levels compared to the CSE-induced group (*p* = 0.0697). There was no significant difference in the TNF-alpha levels of the CSE+L-RBO group when compared with the PBS-induced group (9.47 ± 0.84 vs. 7.75 ± 0.26 pg/mL, *p* = 0.3614), whereas there was a significant difference between the CSE+H-RBO group and the PBS-induced groups (10.60 ± 0.58 vs. 7.75 ± 0.26 pg/mL, *p* = 0.0231).

### 3.5. Effect of RBO on the Levels of MDA in CSE-Induced Emphysema in Mice

**Figure 6:**
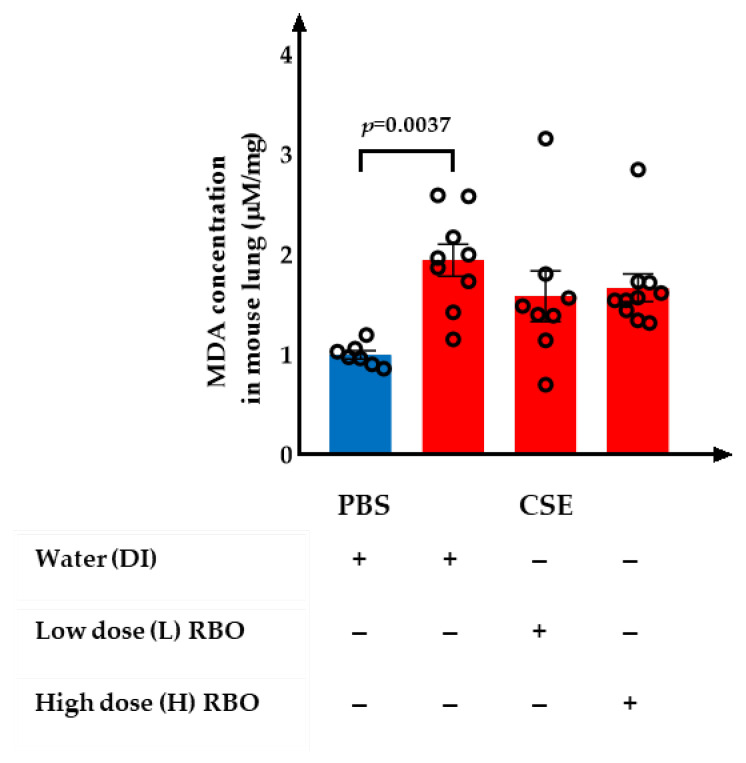
Effect of RBO on the levels of MDA in mouse lung homogenate. All values are presented as mean ± SEMs (*n* = 7–10). One-way ANOVA (Bonferroni’s post hoc test) was used to evaluate the differences among groups with *p*-values for each pair-wise comparison. PBS, phosphate-buffered saline; CSE, cigarette smoke extract; DI, distilled water; L-RBO, low dose of rice bran oil; H-RBO, high dose of rice bran oil; MDA, malondialdehyde.

The quantity of MDA levels in mouse lung homogenate in each experimental group is shown (Figure 6). The levels of MDA were significantly higher in the CSE+DI group than in the PBS+DI group (*p*= 0.0037). The MDA level in the CSE+L-RBO and CSE+H-RBO groups was not significantly different from the CSE+DI group.

### 3.6. Effect of RBO on the Total Antioxidant Capacity in BALF and Serum of CSE-Induced Emphysema in Mice

**Figure 7:**
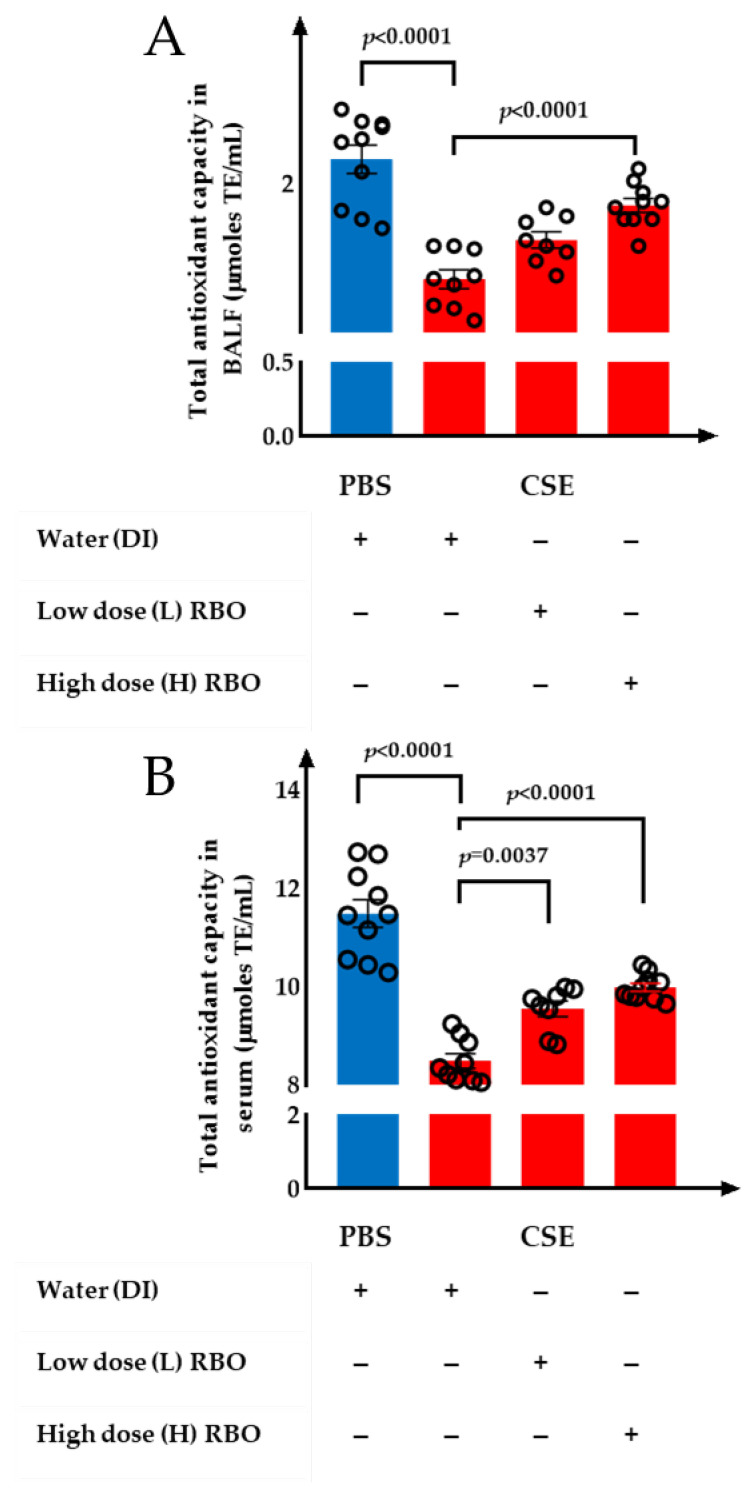
Effect of RBO on total antioxidant capacity in mouse (**A**) BALF samples and (**B**) serum. All values are presented as mean ± SEMs (*n* = 8–10). One-way ANOVA (Bonferroni’s post hoc test) was used to evaluate the differences among groups with *p*-values for each pair-wise comparison. PBS, phosphate-buffered saline; CSE, cigarette smoke extract; DI, distilled water; L-RBO, low dose of rice bran oil; H-RBO, high dose of rice bran oil; BALF, bronchoalveolar lavage fluid.

The total antioxidant capacity in BALF and serum was analyzed using ORAC assay. The total antioxidant capacity in BALF of the CSE+DI group was significantly reduced compared to the PBS+DI group (*p* < 0.0001) (Figure 7A). In comparison with the CSE+DI group, the total antioxidant capacity in BALF of the CSE+L-RBO group did not present a significant difference (*p* = 0.119). However, the CSE+H-RBO group had a significantly higher total antioxidant capacity in BALF than the CSE+DI group (*p* < 0.0001).

The total antioxidant capacity in serum is shown in Figure 7B. The total antioxidant capacity in serum was higher than that in BALF (*p* < 0.0001). The CSE+DI group showed a significant decrease in the total antioxidant capacity in serum compared with the PBS+DI group (*p* < 0.0001). The total antioxidant capacity in serum of the CSE+L-RBO and CSE+H-RBO groups was significantly higher than the CSE+DI group (*p* = 0.0037 with CSE+L-RBO and *p* < 0.0001 with CSE+H-RBO).

## 4. Discussion

In the present study, we investigated the antioxidative and anti-inflammatory effects of RBO containing gamma-oryzanol in a CSE-induced emphysema mice model via oral gavage feeding for consecutive 14 days. RBO was absorbed and circulated to local organs of the experimental mouse. There was no report on clinical abnormality and mortality after RBO administration of < 40 mL/kg mouse body weight (equivalent to <0.8 mL/20 g mouse body weight) [33]. Similarly, no clinical abnormality was observed in this study. Our results demonstrated that RBO administration (0.25 mL and 0.5 mL/20 g mouse body weight) did not significantly affect body weight and weight gain in the CSE-induced mouse emphysema model. The results suggest that RBO did not affect the body weight in CSE-induced emphysema in mice.

Lung inflammation is one of the main mechanisms for chronic obstructive pulmonary disease (COPD) development, causing abnormalities of airways and alveolar walls in patients [34]. Exposure to cigarette smoke, a major risk factor for the development of COPD, induces lung inflammation by continuously releasing inflammatory cells into lung tissue, especially macrophages and neutrophils [35]. An increase in macrophages, neutrophils, and related mediators promotes airway and alveolar epithelium destruction in acute exacerbated COPD patients [36,37]. These inflammatory cells release pro-inflammatory cytokines to amplify and increase the inflammatory response in the lung. TNF-alpha is one of the pro-inflammatory cytokines which is produced and secreted by monocytes/macrophages [38]. An increase in macrophages after exposure to cigarette smoke is strongly related to an increase in TNF-alpha levels in COPD. Inflammatory cells and pro-inflammatory cytokines were commonly detected in the mouse BALF sample after exposure to cigarette smoke extract. Therefore, an increase in inflammatory cells and TNF-alpha levels in the BALF sample indicates inflammation in the lung tissue. The significant findings of this study highlight that the administration of RBO reduced total inflammatory BALF cells, macrophages, neutrophils, and TNF-alpha levels in CSE-induced mice when compared with the CSE-only group, indicating the anti-inflammatory effect of RBO in a CSE-induced emphysema mouse model. RBO was reported to regulate inflammation by suppressing the activation of macrophages and pro-inflammatory cytokines by gamma-oryzanol [39]. Gamma-oryzanol is one of the main active ingredients in rice bran extract [40]. Gamma-oryzanol significantly suppressed the NF-kB activity in colitis mice when compared with a normal food diet. Gamma-oryzanol in rice bran was suggested to have the ability to inhibit inflammatory response by disturbing the activation and nuclear translocation of NF-kB. A significant decrease in mRNA expression of colon TNF-alpha, IL-1, and IL-6 was dominantly observed in dextran sulfate sodium-induced colitis in mice with a gamma-oryzanol diet (100 mg/kg body weight/day for 18 days) when compared with a normal food diet [41,42]. In addition, the gamma-oryzanol diet significantly decreased the activity of colon myeloperoxidase, an enzymatic biomarker for neutrophilic granulocytes, in dextran sulfate sodium-induced colitis in mice when compared with a normal food diet. Therefore, the alteration of total inflammatory BALF cells, macrophages, neutrophils, and TNF-alpha levels positively reflects the anti-inflammatory effect of RBO. This study demonstrated that only a low dose of RBO showed a significant ability to decrease total inflammatory BALF cells and macrophages after CSE exposure. Rice bran has been found to have immunoregulatory activity [43]. Dietary RBO (5% of RBO in total diet/day for 2 weeks) increased lymphocyte numbers due to an improved immune response in a male broiler chicken model [44]. Therefore, an increase in BALF macrophages of the high-dose-RBO-treated group might involve in the overactive effect of immune response due to the over-consumption of RBO in mice. The dose of RBO in this study is converted from a figure of one-fold and two-fold daily dietary fat consumption of 60 kg bodyweight Thai people (55 g and 110 g/day, respectively). The Thai recommended daily intake (Thai RDI) of dietary fat consumption is no more than 65 g [45]. The high dose of RBO was considered as an overdose for dietary consumption of fat. Therefore, the amount of RBO needs to be studied to observe dose response in CSE-induced emphysema in mice.

Oxidative stress is an imbalance between the overproduction of free radicals and the reduction in antioxidant activity [46]. Exposure to cigarette smoke strongly generates the overproduction of free radicals, causing lipid peroxidation from oxidative stress in the lung [47]. MDA is a biological product of lipid peroxidation in oxidative stress [48]. Therefore, an increase in MDA levels represents the lipid peroxidation from oxidative stress in COPD. Serum MDA was significantly higher in smokers than non-smokers [49,50]. However, analysis of the results showed that CSE with RBO treatment has no significant difference in MDA levels compared to the CSE-only group. The antioxidative properties of rice bran have been reported in several animal models [51,52]. Tocotrienols, tocopherols, and gamma-oryzanol are important antioxidant agents in rice bran extract, leading to the suppression of oxidative damage [42]. Correspondingly, treatment with RBO (0.4 mL/day for 5 weeks) significantly reduced serum MDA levels in haloperidol-induced tardive dyskinesia in rats when compared with the non-treated group [53]. Conversely, our results found that RBO did not have any significant effects on MDA levels in mouse lung homogenate. This study was designed to observe the acute effect of RBO after administration for 14 days [33]. The numbers of inflammatory BALF cells were altered in mice with RBO administration for 14 days when compared with the control group. Thus, the duration of RBO administration needs to be studied to observe the alteration of MDA levels in mouse lung. Previous studies demonstrated the histopathological alteration of acute lung injury in humans using electron microscopy [54]. Electron microscopy (EM) is a research technique for investigating the structure of cells, tissues, and organelles using high-resolution images. The EM technique can be applied in further studies to observe the histological change in alveolar walls in mouse lungs after CSE-induced airspace enlargement.

In addition, an increase in oxidative stress is related to a decrease in antioxidant activity [55]. A significant reduction in serum Trolox-equivalent antioxidant capacity with an increase in MDA levels was found in smokers after comparing with non-smokers [56]. Exposure to cigarette smoke (2 cigarettes/15 min/time, 2 times/day, 6 days/week for 16 weeks) significantly decreased the levels of enzymatic antioxidants (superoxide dismutase, glutathione peroxidase, and catalase) and non-enzymatic antioxidants (vitamin E and C) in mouse serum when compared with the control group [57]. CSE administration (0.3 mL/20 g mouse body weight 3 times on days 1, 12, and 23 of the experiment) significantly decreased the serum levels of superoxide dismutase in mice when compared with the control group [26]. Correspondingly, CSE significantly decreased total antioxidant capacity in mouse BALF and serum compared to the control group, indicating the effect of i.p. CSE injection on a reduction in antioxidants of local and systemic fashions in this study. Thus, an increase in MDA levels with a decrease in total antioxidant capacity in this study strongly represents the oxidative stress in mouse lungs. Moreover, there was no significant difference in serum levels of superoxide dismutase between inhalation of cigarette smoke and i.p. CSE injection-induced emphysema in mice [26]. Hence, the route of administration did not have any effect on the antioxidant levels. Our results showed that the administration of RBO significantly increased the total antioxidant capacity in mouse BALF and serum of CSE-induced mice when compared with CSE in the non-RBO treated group, indicating improved antioxidants in the CSE-induced emphysema mouse model after 14-day RBO administration. As mentioned above, RBO exhibited antioxidative potential, which might be associated with bioactive components in RBO. Gamma-oryzanol and vitamin E compounds are major bioactive phytochemicals in rice bran oil [58]. These bioactive compounds in rice bran oil have the ability to enhance the enzymatic antioxidants in an animal model. A previous study reported the antioxidative effect of rice bran oil on arsenite-induced oxidative stress in rats [59]. Exposure to arsenite (As) is known to promote the overuse failure of antioxidant enzymatic defense especially in the liver and brain due to the overexpression of free radicals. The study reported that the administration of rice bran oil (10 mg/kg body weight for 14 days) significantly increased the activity of antioxidant enzymes; SOD, CAT, and GPx, in the liver, brain, and erythrocyte membrane in arsenite-induced oxidative stress in rats when compared with the control group [59]. Correspondingly, our study revealed that 14-day treatment with rice bran oil significantly increased the local and systemic antioxidant capacity of the CSE-induced emphysema mouse model.

Enlarged airspace is a morphometric change in the lung due to the destruction of alveolar walls after exposure to cigarette smoke [60]. The destruction of alveolar walls, a major characteristic of emphysema, is related to an increase in lung inflammatory cells. The protease enzymes are released for those inflammatory cells, causing the degradation of alveolar walls in the lung. Therefore, exposure to cigarette smoke powerfully induces airspace enlargement in the lung. In this study, the cigarette smoke extract (CSE) is collected from mainstream cigarette smoke, containing total particulate matter and several components without the gas from smoke [61]. Introducing CSE into a mouse intraperitoneally can result in airspace enlargement with a reduction in alveolar walls in mouse lung, a main characteristic of emphysema, by environmental exposure. However, intraperitoneal CSE injection is not consistent with the human smoking pattern and the mechanism of COPD development in humans. Additionally, it was reported that mice and rat models with CSE and CS exposure did not demonstrate the pathology in small airways, including goblet cell metaplasia and increased mucus expression as presented in human COPD lung pathology [61]. Therefore CSE- and CS-induced animal models of pulmonary emphysema reflect only some COPD pathologies.

In this study, lung microscopic investigations and MLI analysis were carried out to evaluate the effects of RBO treatment on lung morphology and airspace enlargement in CSE-induced emphysema in mice. The results demonstrated that RBO did not decrease MLI in CSE-induced mice when compared with the CSE-only group. Previous studies reported that the alveolar abnormality is poorly reversible due to the disruption of alveolar regeneration after exposure to cigarette smoke [62]. Our study revealed that RBO can inhibit the severity of alveolar destruction in mouse lungs after i.p. CSE injection by suppressing the number of total inflammatory cells, macrophages, neutrophils, and TNF-alpha levels in the BALF sample. Similarly, the administration of gamma-oryzanol inhibited the inflammatory response by decreasing the pro-inflammatory cytokines and influx of inflammatory cells in dextran sulfate sodium-induced colitis in mice when compared with a normal food diet [41]. Our study revealed that 14-day RBO administration did not significantly affect CSE-induced airspace enlargement in mice. Even though RBO treatment can decrease the levels of inflammatory cells, pro-inflammatory cytokines, and MDA, such decreases did not reach the baseline. Thus, some degrees of lung inflammation and oxidative stress still took place and perhaps prevented the significant effect of RBO administration on CSE-induced airspace enlargement. Therefore, airspace size was not changed in this study. However, the dose–response of RBO in the emphysema mouse model needs to be clarified in further studies in CSE-induced emphysema in mice to observe its anti-inflammatory and antioxidative properties. For clinical implication, the anti-inflammatory effect of RBO could be a new potential therapeutic option to relieve the severity of COPD. The present study analyzed the effects of RBO after emphysema. It would be very interesting to investigate the preventive effects of RBO before emphysema is induced in the next study.

## 5. Conclusions

This study demonstrated that RBO can suppress local lung inflammation by reducing total inflammatory cell, macrophage, and neutrophil numbers and TNF-alpha levels in BALF and by lessening the airspace size of CSE-treated mice, suggesting the anti-inflammatory effect of RBO on CSE-induced emphysema in a mouse model. In addition, RBO exhibited antioxidative potential on CSE-induced emphysema mice by increasing the total antioxidant capacity in BALF and serum. Therefore, for clinical implications, the anti-inflammatory and antioxidative effects of RBO could be a new potential therapeutic option to relieve the severity of COPD.

## Data Availability

The datasets generated for this study are available on request from the corresponding author.
